# Quality Assessment of Gallbladder Cancer Pathology Reports: A Dutch Nationwide Study

**DOI:** 10.3390/cancers13122977

**Published:** 2021-06-14

**Authors:** Tessa J. J. de Bitter, Elise A. J. de Savornin-Lohman, Philip R. de Reuver, Valerie Sophie Versteeg, Elisa Vink-Börger, Joanne Verheij, Iris D. Nagtegaal, Rachel S. van der Post

**Affiliations:** 1 Department of Pathology, Radboud University Medical Center, 6500 HB Nijmegen, The Netherlands; V.Versteeg@student.ru.nl (V.S.V.); Elisa.Vink-Borger@radboudumc.nl (E.V.-B.); Iris.Nagtegaal@radboudumc.nl (I.D.N.); Chella.vanderPost@radboudumc.nl (R.S.v.d.P.); 2 Department of Surgery, Radboud University Medical Center, 6500 HB Nijmegen, The Netherlands; Elise.deSavorninLohman@radboudumc.nl (E.A.J.d.S.-L.); Philip.deReuver@radboudumc.nl (P.R.d.R.); 3 Department of Pathology, Cancer Center Amsterdam, Amsterdam UMC, University of Amsterdam, 1105 AZ Amsterdam, The Netherlands; j.verheij@amsterdamumc.nl

**Keywords:** pathology report, completeness, surgical pathology, narrative

## Abstract

**Simple Summary:**

Appropriate reporting of pathological findings is required for optimal patient care and to perform high-quality research. The aim of our study was to assess the completeness of pathology reports of gallbladder cancer (GBC) in a large nation-wide patient cohort from the Netherlands. Results showed that reports were often incomplete; information on essential items that can predict prognosis were often missing. Whereas certain items were often missing in the report, they could be retrospectively detected in a large proportion of patients during pathology review. Our findings showed that significant improvements could be made in the reporting of gallbladder cancer in the Netherlands. To this end, the added value of standardized (synoptic) reporting should be explored, of which the beneficial effects have already been demonstrated in other tumor types.

**Abstract:**

Adequate reporting of pathological findings is essential for optimal patient management and to perform high-quality research. The aim of this study was to assess the completeness of pathology reports of gallbladder cancer (GBC) at the nationwide level to assess guideline adherence and make recommendations for improvement. A retrospective population-based cohort of GBC patients diagnosed in the Netherlands from 2000 to 2019 was collected using data from the Dutch Cancer Registry and the nationwide network and registry of histology. Pathology reports were scored on the presence and content of essential and optional items according to the Dutch consensus-based guideline on biliary tract cancer. By histopathological review of cases, we compared findings with the conclusion of the corresponding pathology report. All pathology reports (*n* = 849) had a narrative, nonstructured format. Overall completeness was low. Information on key prognostic factors, such as tumor side (hepatic vs. serosal), status of cystic duct and liver surgical margins and venous and perineural invasion, was frequently lacking (80%, 23%, 59%, 74% and 74% missing, respectively). Whereas certain items were often missing from the report, they could be retrospectively detected in a substantial proportion of cases during pathology review (*n* = 738). In conclusion, significant improvements could be made in the reporting of GBC in the Netherlands. Synoptic reporting could greatly enhance the completeness of reports, as already demonstrated for tumor types.

## 1. Introduction

Gallbladder cancer (GBC) is a rare malignancy, especially in Western countries, and notoriously lethal due to its aggressive behavior and the absence of effective treatment options [1]. In 15–20% of patients, GBC is discovered incidentally (iGBC). IGBC is diagnosed either perioperatively or postoperatively after cholecystectomy for benign gallbladder disease (e.g., symptomatic cholecystolithiasis or cholecystitis) (12623362). Especially in patients with iGBC, adequate reporting of pathological findings is imperative for clinical decision making as patients with muscle-invasive GBC (pT1b and higher) will benefit from radical reresection [2]. Precise staging is dependent on the reporting of multiple items in the pathology reports, such as the side of the tumor (hepatic or serosal side), depth of invasion (T-stage) and status of resection margins.

Among the challenges in diagnosing and treating a rare cancer such as GBC is that pathological expertise is scarce. A lack of volume and expertise may lead to an incomplete assessment of the resection specimen, or the reporting thereof, with possible erroneous staging and suboptimal treatment as a consequence. Chatelain et al. previously demonstrated that pathological reports of iGBC cases are frequently incomplete [3]. Essential information on factors, such as location of the tumor, depth of infiltration, surgical margins, tumor differentiation and venous, lymphatic and perineural invasion, was often not present. Furthermore, the majority of reports (93%) lacked any standardization. As only iGBC cases were included in the study of Chatelain et al. [3], the completeness of pathology reports of nonincidental GBC cases remains unknown.

The Dutch consensus-based guideline on biliary tract cancer (last update: 20 May 2013, [4]), advises standardized reporting for GBC and includes a list of essential and optional items to be present in the pathology report. Globally, there is an increased trend towards the standardization of pathology information, through initiatives such as the International Collaboration on Cancer Reporting (ICCR). The ICCR published several digestive tract datasets, including intrahepatic cholangiocarcinoma, perihilar cholangiocarcinoma and hepatocellular carcinoma [5,6]. For GBC, however, a standardized ICCR dataset is currently lacking. The College of American Pathologists (CAP) published a gallbladder cancer reporting protocol that requires more extensive, standardized reporting than the current Dutch consensus guideline [7].

In this study, we aimed to provide an overview of the completeness of pathology reports on GBC in the Netherlands to assess adherence to the Dutch guideline before and after the update in 2013. Furthermore, when available, cases were reviewed to compare findings with those in the pathology report to provide recommendations for improvement.

## 2. Materials and Methods

### 2.1. Patient Selection

By combining databases of the Netherlands Cancer Registry (NCR, k171236) and the nationwide network and registry of histo- and cytopathology (PALGA, LZV2017-87), we identified patients with GBC between 2000 and 2019 in the Netherlands. Next, complete anonymized pathology reports were sent to us upon request by participating pathology labs.

### 2.2. Review of Pathology Reports

All reports were examined on the presence and content of essential and optional items of the pathology report according to the Dutch guideline on biliary tract cancer (last update: 20 May 2013 [4]). Essential items include histological type; histological grade; TNM stage (including total number of resected and affected lymph nodes); resection margins of liver and cystic duct (including distance to the resection plane in case of negative margins); and venous, lymphatic and perineural invasion. In addition to the essential items, optional items include the diameter of the tumor; localization of the tumor; macroscopic appearance; and other pathological findings, such as dysplasia, cholelithiasis and chronic cholecystitis.

### 2.3. Review of Histopathology

Cases for which hematoxylin- and eosin (H&E)-stained slides were available were reviewed by a pathology team (E.V.B. and R.S.V.D.P.) according to the same guideline. Next, findings were compared with those present in the pathology report for binary variables: lymphatic, venous, and perineural invasion to determine the extent of deviation from the report and to provide recommendations for improvement.

### 2.4. Outcomes

The primary outcome of this study was the completeness of pathology reports, scored as the percentage of included items as recommended by the Dutch guideline. Completeness was compared for reports before and after the update of this guideline in 2013.

Secondary outcomes were the comparison of completeness between academic and nonacademic centers, and the comparison of the presence or absence of lymphatic, venous and perineural invasion as reviewed by the pathology team, compared to the outcome of the pathology report.

### 2.5. Statistics

Completeness of items was described using counts and percentages. The Pearson’s chi-squared (Χ^2^) test was used to compare dichotomous variables. Statistical tests were performed using Graphpad Prism, version 5.03 (GraphPad, San Diego, CA, USA). Two-sided tests were performed, and a *p* value < 0.05 was considered statistically significant. Sankey diagrams were created using SankeyMATIC [8].

## 3. Results

### 3.1. Demographic Characteristics

In total, 849 pathology reports of incidental and nonincidental GBC, from a period of 2000–2019, were analyzed. The reports were derived from 33 pathology laboratories, including 6 academic hospitals and 27 general hospitals or diagnostic service pathology laboratories spanning more than one general hospital. The median number of reports per laboratory was 21 (range: 4–70 reports). All reports had a narrative, nonstructured format. Of 738 resection specimens, either original H&E stained slides or tissue blocks to recut slides were available for histopathological review.

### 3.2. Overall Completeness of the Reports

Table 1 summarizes the overall completeness of 849 reports for the essential and optional (marked with an asterisk) items of the pathology report according to the Dutch guideline. None of the reports included information on all items. Information on clinical parameters, provided by the surgeon, including the type of surgical procedure, clinical indication and administration of neoadjuvant chemotherapy was complete in 43.8%, 86.3% and 0.1% of reports, respectively.

Gross macroscopic features, including tumor location, tumor side (hepatic vs. serosal), tumor size and macroscopic appearance of tumor, are optional items and were reported in less than half of the cases. Microscopic features, including histologic type, T-stage, tumor grade and cystic duct margin, were adequately reported (from 77.4% to 99.5% complete for cystic duct margin and histologic type, respectively). The subclassification of stage T2a and T2b was first introduced in the current TNM staging edition (8th edition, 2018). Hence, for the majority of reports, this was not a required item to be reported at the time of diagnosis, and the subclassification of T2a and T2b was reported in 99/385 (25.7%) of T2 patients. All other microscopic features were reported in less than half of the cases, including the liver resection margin in only 41.1% of cases and distance to noninvolved cystic duct and liver margins in 10.2% and 27.5% of cases, respectively. Histologic subtype, such as subtype of adenocarcinoma (e.g., intestinal versus biliary type), is not a required item and was reported only in a few cases.

After the introduction of the Dutch consensus guideline in 2013, a significant increase in completeness was observed for nearly all items. Improvement was not significant for histologic type, pT1a vs. pT1b and tumor grade, but completeness was already high for all three items before the introduction of the guideline. Furthermore, pathology reports from academic hospitals were significantly more complete than pathology reports from general hospitals, for the majority of items. This was irrespective of the number of reports per hospital for the general hospitals.

### 3.3. Comparison between Pathology Report and Pathology Review

**Figure 1:**
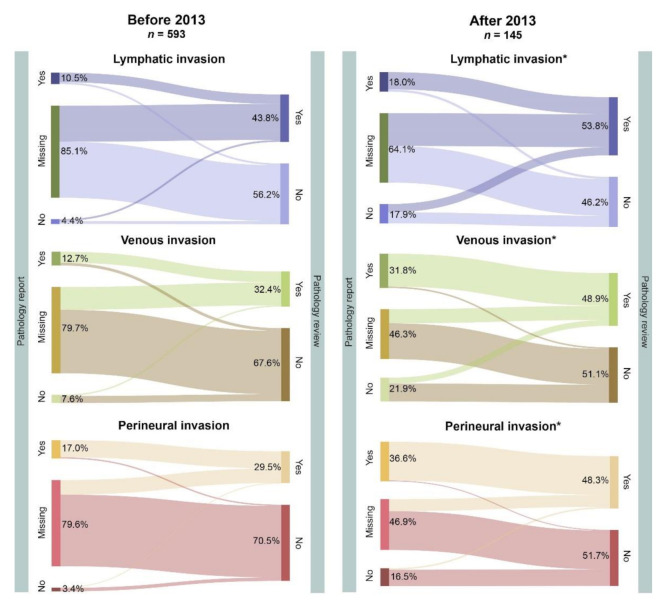
Comparison of pathology reports with pathology review before and after the update of the Dutch consensus guideline in 2013. For 738/849 cases, histopathological characteristics were reviewed by a pathology team, and results were compared with those from the report. In a large proportion of cases, data were missing from the original pathology report. A significant increase in concordance (report and review both “Yes” or both “No”) for all three types of invasion was observed after introduction of the consensus guideline in 2013, mainly due to a decrease in missing values. Results were analyzed by two-tailed Pearson’s Χ^2^-test. *: *p* < 0.05.

Cases were reviewed according to the Dutch guideline; for binary variables, lymphatic, venous and perineural invasion, findings were compared with those of the original pathology report (Figure 1). In a substantial proportion of pathology reports, data were missing on all three types of invasion. After the implementation of the new guideline in 2013, the proportion of missing items decreased, leading to a significant increase in concordance between the pathology report and pathology review. For perineural invasion, the majority of the missing data were scored as absent during pathology review, while for lymphatic and venous invasion, the missing data were more randomly distributed to presence and absence during pathology review. When nonmissing data were excluded, the discordance between pathology report and pathology review was low.

## 4. Discussion

This study showed that the current practice for histopathology reporting of cholecystectomy specimens with GBC in the Netherlands lacks any standardization and that reports are often incomplete. Even essential items for staging, which include depth of invasion (T-stage) and nodal status, were incomplete in a substantial number of reports. Tumor side (hepatic vs. serosal) was reported in less than half of the cases. Moreover, there was significant discordance between the presence and absence of certain histopathological characteristics when comparing the histopathology reports to histopathological review.

Even though a significant increase in completeness was observed after the update of the guideline in 2013, reports were still often lacking important prognostic factors, such as tumor side (hepatic vs. serosal). Whereas this is still an optional item in the current national guideline, from 2018 onwards, the most recent international TNM classification (TNM8) requires a distinction between T2a (serosal side) and T2b (hepatic side) for all T2 cases. When considering all T2 patients, including those from before and after 2018, a distinction between T2a and T2b was made in merely 25.7% of patients. This distinction is of particular importance, since tumor side is a strong predictor of prognosis in T2 GBC [9], and is important in the selection of patients that benefit from radical re-resection [9,10,11,12].

In general, missing data on specific features are grouped under negative or absent [13]. We showed that this assumption is false. After review, 19.5% to 32.8% of all cases showed the presence of venous and lymphatic invasion, respectively, whereas these items were absent from the report. In contrast, for perineural invasion, most missing cases corresponded to the actual absence of this feature. Thus, it seems that the presence of perineural invasion is more notable than vascular invasion.

Reports from academic pathology labs were significantly more complete than those from general hospitals. This might partly be explained by the fact that patients with a preoperative suspicion for GBC are more likely referred to academic hospitals that perform more extensive resections than a simple cholecystectomy and, therefore, provide a more detailed report. On the other hand, reports from high-volume general hospitals that also perform more extensive resections were not significantly better than the average. This difference might be explained by the presence of dedicated hepatobiliary pathologists in academic hospitals. Additionally, items that are not of direct clinical relevance might more easily be omitted from a narrative report format, especially in a nonacademic setting, where reports are more aimed to answer questions of direct clinical relevance rather than listing other potentially relevant items.

Improvements could be made by the introduction of synoptic reporting (SR), for which the added value was extensively reviewed by Sluijter et al. [14] and recently confirmed by Baranov et al. [15]. Fourteen included studies measured the effect of SR on the overall completeness of the pathology report [14], of which 13 demonstrated an increased overall completeness after the introduction of SR, independent of cancer type. In addition to improved reporting, the introduction of SR significantly reduced the time spent on preparation of the report [16,17]. The CAP guideline for the examination of GBC specimens could greatly enhance the implementation SR for GBC in the Netherlands [7].

Additional improvements could be made by organizing regional multidisciplinary team meetings, to discuss rare GBC patients and their treatment possibilities. At the moment, GBC care is not centralized in the Netherlands, reflected by the large number of pathology laboratories that provided reports for this study and the low median number of reports annually per laboratory. Since a significant number of GBCs are incidentalomas, GBC can never be fully centralized. In recent years, a Dutch multidisciplinary GBC collaborative was established to discuss clinical decision making for GBC and to increase coherence of GBC research.

To our knowledge, this study describes the completeness of pathology reports of the largest nationwide cohort of incidental and nonincidental GBC patients in the literature. Results are largely in line with those from Chatelain et al. [3], who also showed frequent incompleteness, albeit solely in iGBC. We additionally reported differences after the implementation of a nationwide guideline and compared academic hospitals with general hospitals. Future studies could be directed towards the comparison of SR and narrative reporting in GBC.

## 5. Conclusions

In conclusion, significant improvements could be made in the reporting of GBC in the Netherlands. To this end, the added value of SR in GBC is presumably high. Adequate reporting of pathological findings is imperative for optimal diagnosis, prediction of prognosis and patient care. Moreover, increased completeness will not only benefit the satisfaction among end-users and support clinical decision making, but is also invaluable to perform high-quality research.

## Figures and Tables

**Table 1:** Completeness of GBC pathology reports.

	All (*n* = 849)	<2013 (*n* = 684)	>2013 (*n* = 165)	*p*-Value	Academic (*n* = 143)	Non-Academic (*n* = 706)	*p*-Value
	No. of Cases Reported (%)	No. of Cases Reported (%)	No. of Cases Reported (%)		No. of Cases Reported (%)	No. of Cases Reported (%)	
Clinical parameters							
Procedure	372 (43.8%)	277 (40.5%)	95 (57.6%)	**<0.0001**	92 (64.3%)	280 (39.7%)	**<0.0001**
Indication	733 (86.3%)	582 (85.1%)	151 (91.5%)	**0.0310**	136 (95.1%)	597 (84.6%)	**0.0008**
Neoadjuvant treatment	1 (0.1%)	0 (0.0%)	1 (0.6%)	**0.0416**	1 (0.7%)	0 (0.0%)	**0.0262**
Gross macroscopic features *							
Tumor location	283 (33.3%)	209 (30.6%)	74 (44.8%)	**0.0005**	77 (53.8%)	206 (29.2%)	**<0.0001**
Tumor side (hepatic vs. serosal)	173 (20.4%)	120 (17.5%)	53 (32.1%)	**<0.0001**	57 (39.9%)	116 (16.4%)	**<0.0001**
Macroscopic appearance	268 (31.6%)	200 (29.2%)	68 (41.2%)	**0.0030**	61 (42.7%)	207 (29.3%)	**0.0018**
Tumor size	357 (42.0%)	264 (38.6%)	93 (56.4%)	**<0.0001**	93 (65.0%)	264 (37.4%)	**<0.0001**
Microscopic features							
Histologic type	845 (99.5%)	682 (99.7%)	163 (98.8%)	0.1215	143 (100%)	702 (99.4%)	0.3669
T-stage	737 (86.8%)	583 (85.2%)	154 (93.3%)	**0.0058**	130 (90.9%)	607 (86.0%)	0.1120
T1a vs. T1b (where applicable) ^a^	104/107 (97.2%)	83/86 (96.5%)	21/21 (100%)	0.3853	16/16 (100%)	88/91 (96.7%)	0.4613
N-stage	298 (35.1%)	216 (31.6%)	82 (49.7%)	**<0.0001**	89 (62.2%)	209 (29.6%)	**<0.0001**
Tumor grade	636 (74.9%)	507 (74.1%)	129 (78.2%)	0.2804	93 (65.0%)	543 (76.9%)	**0.0028**
Cystic duct margin	657 (77.4%)	517 (75.6%)	140 (84.8%)	**0.0107**	111 (77.6%)	546 (77.3%)	0.9407
Distance to resection plane reported ^b^	45/440 (10.2%)	21/333 (6.3%)	24/107 (22.4%)	**<0.0001**	16/76 (21.1%)	29/364 (8%)	**0.0006**
Liver margin	349 (41.1%)	258 (37.7%)	91 (55.2%)	**<0.0001**	89 (62.2%)	260 (36.8%)	**<0.0001**
Distance to resection plane reported ^b^	58/211 (27.5%)	34/152 (22.4%)	24/59 (40.7%)	**0.0075**	28/89 (31.5%)	30/122 (24.6%)	0.2696
Lymphatic invasion	157 (18.5%)	97 (14.2%)	60 (36.4%)	**<0.0001**	42 (29.4%)	115 (16.3%)	**0.0002**
Venous invasion	218 (25.7%)	132 (19.3%)	86 (36.4%)	**<0.0001**	62 (43.4%)	156 (22.1%)	**<0.0001**
Perineural invasion	219 (25.8%)	136 (19.9%)	83 (36.4%)	**<0.0001**	64 (44.8%)	155 (22%)	**<0.0001**

* Gross macroscopic features including location, side, macroscopic appearance and size of the tumor are optional items in the Dutch consensus guideline. ^a^ The 8th edition of the AJCC staging system was introduced in 2018. Since our cohort only includes very few cases from 2018 onwards, we cannot draw conclusions on the rate of reporting differentiation between T2a and T2b. However, tumor side (hepatic vs. serosal) was reported in 99/385 (25.7%) of T2 patients. ^b^ Reporting of the distance to the resection plane is only required in case of noninvolved margins. For the cystic duct margin, 440/657 patients in whom the margin was reported had noninvolved margins. For the liver margin, 211/349 patients in whom the margin was reported had noninvolved margins. Results were analyzed by two-tailed Pearson’s Χ^2^-test, with a *p*-value of <0.05 (in bold) considered as statistically significant.

## Data Availability

The data presented in this study are available in the article.
